# Nasal High-Flow Oxygen Therapy in Chronic Respiratory Failure for Homecare Applications—A Feasibility Study

**DOI:** 10.3390/jcm13154525

**Published:** 2024-08-02

**Authors:** Achim Grünewaldt, Gernot Rohde

**Affiliations:** Department of Respiratory Medicine and Allergology, University Hospital, Goethe University, 60590 Frankfurt, Germany

**Keywords:** high-flow oxygen, homecare, respiratory failure, interstitial lung disease, cystic fibrosis

## Abstract

**Background:** While high-flow nasal cannulas (HFNCs) represent the standard of care in the intensive care unit for patients with severe hypoxemia, its use in homecare settings is uncommon despite its potential. The potential benefits and challenges of the high-flow nasal cannula (HFNC) in homecare settings compared to standard long-term oxygen via nasal low-flow therapy are unclear. **Methods:** We conducted a prospective monocentric feasibility study at the Department of Respiratory Medicine, University Hospital, Goethe University Frankfurt, Germany. Patients with interstitial lung disease or severe bronchiectasis (including cystic fibrosis) were enrolled into the study. The HFNC was introduced during hospitalization. The patients’ compliance with home use advice and arterial blood gas results were evaluated at a 4–6-week follow-up. **Results:** A total of 12 patients were analyzed. HFNC initiation did not result in a significant improvement of the pO_2_/fiO_2_ (p/f) ratio. Only 8 out of 12 (66.6%) patients used the HFNC at home after the initial in-hospital initiation. Only 7 of the total 12 patients were using the therapy at a follow-up 3–6 weeks after HFNC onset. Two patients died during the observation, resulting in a surveillance mortality rate of 16.7%. **Conclusions:** The feasibility data showed low adherence to the HFNC at home. The lack of any positive effect on the p/f ratio may be due to low airflow rates and overall mild hypoxemia compared to patients with severe respiratory failure in the ICU.

## 1. Introduction

Structural lung diseases, such as interstitial lung disease or severe bronchiectasis, are often associated with progressive hypoxemia, leading to dependence on long-term oxygen therapy (LTOT). Most guidelines recommend the prescription of LTOT when pO_2_ falls below 55 mmHg or, in the case of heart failure, below 60 mmHg [1].

Despite its recognized role in hospitalized patients, the evidence of any beneficial effects of LTOT is limited. Furthermore, most studies have focused on COPD patients with chronic hypoxemia [2].

LTOT has been shown to improve exercise duration in patients with cystic fibrosis [3]. Similar results were published in a meta-analysis by Bell et al. who investigated the effects of oxygen therapy in patients with ILD. The authors reported that studies showed an increase in exercise duration but no improvement in shortness of breath [4].

Unlike conventional low-flow oxygen therapy high-flow oxygen therapy (high flow nasal cannula, HFNC), which uses modified gases, the increased air is heated and humidified. In addition, the inspiratory fraction of oxygen is precisely defined.

In the intensive care setting, HFNCs are the standard of care in patients with severe hypoxemia [5].

Over the last few decades, high-flow application devices for homecare were developed. The possibility of whether these devices influence the quality of life or the outcome of the patients who receive this new therapeutic option has been scarcely examined.

In a recent retrospective study by Ehrlich et al., who examined HFNCs in home settings in pediatric patients, the authors reported that the therapy was well tolerated and that the introduction of the HFNC was associated with fewer hospitalizations [6]. In addition, few case reports showed the sufficient use of the HFNC in palliative homecare settings [7,8].

Therefore, further evidence focusing on HFNCs in homecare settings is warranted.

In the framework of a feasibility study, we examined patients with progressive interstitial lung disease or severe bronchiectasis (including cystic fibrosis) who used LTOT.

We evaluated the effects of HFNCs on respiratory parameters and the patient’s use at home.

The aim of the study was to identify potential issues, such as the patient’s compliance with homecare setting advice and optimal therapy titration, which should be considered when designing large multicentric trials to evaluate HFNCs in homecare settings.

We focused on patients with interstitial lung disease and cystic or non-cystic bronchiectasis because hypoxemic failure is the leading cause of respiratory compromise in both groups.

## 2. Material and Methods

### 2.1. Ethics

The study protocol was approved by the local ethics committee of the Goethe University, Frankfurt, Germany (study number: 291/16; date: 16 September 2016). All patients provided written informed consent.

### 2.2. Patient and Public Involvement

The patients or members of the public were not involved in the design, completion, reporting, or dissemination plans of our research.

### 2.3. Study Design, Subjects, and Primary Endpoints

We conducted a prospective single-center feasibility study at the Department of Respiratory Medicine of the University Hospital Frankfurt/Main, Germany. 

The aim was to collect information on the effectiveness of HFNCs in relation to hypoxemia, the use of HFNCs, and potential difficulties in homecare settings.

The primary endpoint was arterial oxygen pressure before and after HFNC initiation.

From 2016 to 2019, adult patients older than 25 years with interstitial lung disease or bronchiectasis (cystic fibrosis and non-cystic fibrosis) undergoing long-term oxygen therapy with a pO_2_ less than 60 mmHg without oxygen supplementation were enrolled. Patients with severe acidosis (pH < 7.2) or severe hypercapnia (pCO_2_ > 60 mmHg) were excluded.

Patients were enrolled during hospitalization due to the exacerbation of lung disease. In line with the local hospital standards for initiating noninvasive home ventilation, follow-up visits were scheduled up to five weeks after HFNC initiation. The HFNC was initiated with the “TNI soft flow” system (TNI medical AG, Germany). The system provides flowrates between 10 and 50 L/min and humidity levels between 30 and 37° dew points.

We collected sociodemographic and clinical data and compared blood gas changes before and after the initiation of the HFNC.

Therapy parameters were determined by the treating physician. Flow was initiated between 30 and 40 L/min and at a standard humidity level (a dew point of 37°) at the beginning of therapy. The fraction of inspired oxygen was titrated individually according to the oxygen saturation target.

Blood gases were measured before the start of HFNC therapy, after 1–2 h, and 15–24 h after the introduction of the HFNC during oxygen therapy. Another blood gas check was performed during the follow-up visit.

Data were obtained from the electronic medical records of the hospital data system “AGFA-Orbis”. All patients underwent pulmonary function testing during hospitalization.

Data were recorded using a paper case report form and Excel software.

Statistical analysis was performed using the statistical software “SPSS” (IBM SPSS Statistics version 27). Normally distributed data were described with mean and standard deviation (SD) values, and non-normally distributed data were described with median and interquartile range (IQR) values.

The Wilcoxon test was used to examine any differences of oxygenation or decarboxylation before and after the initiation of the HFNC. A *p*-value less than 5% was considered to be significant.

For the secondary endpoint pCO_2_, a treatment effect with an effect size of at least 0.75 kPa could be measured with a sample size of at least 11 patients and 80% power.

## 3. Results

A total of 12 patients with interstitial lung disease or cystic fibrosis were enrolled. Table 1 shows the patient’s diagnoses, age, and gender distribution.

Table 2 gives the sociodemographic and main clinical data, including relevant comedication. During the study period, none of the CF patients were under CFTR modulator therapy.

The median age of the patients was 71 years (IQR: 49.5–75.8 years), and four patients (33.3%) were female. All patients were non-smokers and the mean BMI was 21.8 (SD 3.53).

Five patients (41.7%) were treated with oral steroids before hospitalization and seven patients (58.3%) received oral steroids during hospitalization.

The median C-reactive protein was 1.7 g/dL (IQR 0.47–6.58). No patient had evidence of acute heart failure, and the median nt-proBNP was 210 pg/mL (IQR 91.5–277.9 pg/mL).

Pulmonary function results are summarized in Table 3. Most patients had a severe restrictive pattern on spirometry. The median expiratory forced vital capacity (FVC) was 44% of the predicted normal (IQR: 34.1–54.8%). The forced expiratory volume in one second (FEV1) was 43.9% of the predicted normal (23–58.5%), indicating a severe restrictive pattern in most patients.

Table 4 summarizes the parameters of blood gas analysis before and after the initiation of the HFNC.

The median flow applied was 35 L/min (IQR 26.3–43.8 L/min), with a median oxygen fraction of 31% (IQR 28–55.7%).

Blood gas analysis results showed a mild reduction in oxygenation with a median pO_2_ of 75.3 mmHg (IQR 64–94.7 mmHg). Most patients were not hypercapnic, with a median pCO_2_ of 41.8 mmHg (IQR: 39.1–46.7 mmHg).

The paired Wilcoxon signed rank test showed no significant increase or decrease in the pO_2_/fiO_2_-ratio (p/f ratio) after HFNC initiation. In total, 8 out of 12 patients used the HFNC at home after the initial in-hospital initiation.

Only 7 of the total 12 patients were using the HFNC at the follow-up 3–6 weeks after the initiation.

Two patients died during the follow-up, resulting in a surveillance mortality rate of 16.7%.

No exacerbation of ILD or CF was observed during the follow-up.

## 4. Discussion

This feasibility study was conducted to evaluate the effects and potential difficulties of HFNCs in homecare settings.

All 12 recruited patients had a severe impairment of the pulmonary function and were on long-term oxygen therapy prior to HFNC initiation.

It is remarkable that despite the severe reduction in expiratory forced vital capacity and severe airflow limitation, oxygenation only mildly decreased at rest. One explanation for the initial initiation of LTOT could be an exercise-dependent impairment of oxygenation, which was not part of the study protocol. Another aspect of relevance is the discrepancy between the standardized exercise testing and desaturations in daily life, which may have led to the initial initiation of LTOT [9].

We did not observe a significant improvement in oxygenation or decarboxylation after HFNC initiation during the first follow-up observation at 2 or 24 h. Several factors could explain these results.

The HFNC represents a standard of care in the intensive care treatment of severe hypoxemia. Beyond its use in acute hypoxemic failure, there is some evidence that HFNC can reduce pCO_2_ levels, which may be a result of the reduction in death space and the work of breathing with HFNCs. Accordingly, it may be an alternative treatment of hypercapnia in patients intolerant of noninvasive ventilation [10].

However, most of the studies demonstrating the beneficial effects of HFNCs in the ICU setting have used very high airflow rates of at least 40–50 L/min [11,12]. In our study, the HFNC was established in a non-ICU setting in most cases. The flow was titrated according to patient tolerance and the duration of therapy was not standardized. In addition, the impairment of oxygenation and decarboxylation may have been too small to show the significant effects of HFNCs.

The most important finding was that only about 60% of the patients returned to follow-up and only 67% used the HFNC at home. This demonstrates the importance of patient surveillance after the initiation of any form of home ventilatory support. Similar adherence results were reported by Vosse et al., who studied ventilator adherence in patients with neuromuscular diseases over a one-year follow-up period [13]. The authors published an adherence rate of only 62%. Most of the patients who discontinued the therapy did not perceive any clinical benefit from the therapy.

In a recent study, Volpato et al. demonstrated that a psychological interaction can increase the adherence to and acceptance of noninvasive ventilation at home [14]. Further studies on this topic may involve interventions to increase patients’ use of the therapy.

## 5. Conclusions

This feasibility study did not demonstrate an improvement in oxygenation using HFNC therapy in patients with a mild impairment of oxygenation or decarboxylation. Adherence to the therapy at home was low.

These results underscore the importance of follow-up visits and patient support when using any form of home ventilatory support.

A flow rate below 40 L/min may not have a beneficial effect on oxygenation compared to conventional low-flow oxygen supplementation.

## 6. Limitations

The purpose of our study was to examine the use, adherence, and potential problems of HFNCs at home in a cohort of patients with restrictive lung disease.

Accordingly, the small sample size is not adequate to evaluate the efficacy of the HFNC itself.

The HFNC parameters were titrated by the decision of the treating physician. With this, another problem is the lack of a standardized therapy protocol which could have led to a more effective HFNC titration.

## Figures and Tables

**Table 1:** Distribution of diagnosis; COPD = chronic obstructive pulmonary disease.

Age	Gender	Diagnosis	Cardiovascular Comorbidities
79	female	pleuroparenchymal fibroelastosis	arterial hypertonus
75	male	eosinophilic pneumonia	coronary artery disease, history of pulmonary embolism
41	male	cystic fibrosis	none
76	male	idiopathic pulmonary fibrosis	none
74	female	systemic sclerosis	coronary artery disease, carotid stenosis
33	male	cystic fibrosis	none
44	male	cystic fibrosis	none
67	male	sarcoidosis, COPD	coronary artery disease, cardiac insufficiency
85	female	progressive pulmonary fibrosis	arterial hypertension
70	male	idiopathic pulmonary fibrosis	none
72	male	nonspecific interstitial pneumonia	arterial hypertension
66	female	hypersensitivity pneumonitis/poliomyelitis	none

**Table 2:** Sociodemographic and clinical data; hsCRP = highly sensitive C-reactive protein; IQR = interquartile range; nt-proBNP = n-terminal pro-brain natriuretic peptide.

Sociodemographic and Clinical Data	n Available	Mean (sd)/Median (IQR); n (%)
age; median (IQR) [years]	12	71 (49.5–75.8)
gender, female (n/%)	12	4 (33.3%)
BMI; mean (sd)	12	21.8 (sd 3.53)
height; mean (sd) [cm]	12	170.9 (sd 11.0)
weight; mean (sd) [kg]	12	63.7 (sd 11.5)
active smoking; (n)	12	0
oral steroid therapy before admission; n (%)	12	5 (41.7%)
oral steroid therapy during hospital stay; n (%)	12	7 (58.3%)
bronchodilator therapy; n (%)	12	8 (66.7%)
antibiotic therapy during hospitalization; n (%)	12	6 (50%)
antifibrotic therapy; n (%)	12	3 (25%)
opioid therapy during hospital stay; n (%)	12	0
long-term oxygen therapy before admission; n (%)	12	12 (100%)
hsCRP; median (IQR) [mg/dL] (normal < 0.5)	11	1.7 (0.47–6.58)
nt-proBNP; median (IQR) [pg/mL]	5	210 (91.5–277.9)

**Table 3:** Pulmonary function results. FEV1 = forced expiratory volume in one second; FVC = forced vital capacity; IQR = interquartile range.

Pulmonary Function Test Parameters	n Available	Median (IQR)
FVC; median (IQR) [L]	12	1.71 (1.12–1.94)
FVC % predicted; median (IQR)	12	44.0 (34.1–54.8)
FEV1; median (IQR) [L]	12	1.07 (0.78–1.32)
FEV1 % predicted; median (IQR)	12	43.9% (23.0–58.5)
TLC; median (IQR) [L]	10	2.8 (2.4–4.2)
TLC % predicted value; median (IQR)	10	51.5 (84.4–61.5)
RV; median (IQR) [L]	10	1.5 (1.19–3.46)
RV % predicted; median (IQR)	10	70% (53.4–154.9)
piMax % predicted; median (IQR)	8	74.0 (48.9–116.0)

**Table 4:** Parameters of therapy and outcome; fiO_2_ = fraction of inspired oxygen; IQR = interquartile range; p/f ratio = pO_2/_fiO_2_.

Parameters of HFNC, Blood Gas Results, and Outcomes	n Available	Median (IQR)/n (%)	*p*-Value *
flow; median (IQR) [L/min]	12	35 (26.3–43.8)	
fiO_2;_ median (IQR) [%]	12	31 (28–55.7)	
HFNC use at home; n (%)	12	8/12 (66.7%)	
pO_2_ before start; median (IQR) [mmHg]	12	75.3 (64–94.7)	
p/f ratio before start; median (IQR)	12	236.3 (168.1–302)	
pO_2_ 2 h follow-up; median (IQR) [mmHg]	12	70.6 (62.6–89.7)	0.695
p/f ratio 2 h after follow-up; median (IQR)	11	233.1 (170.6–247.9)	0.374
pO_2_ 24 h after follow-up; median (IQR) [mmHg]	9	76.3 (62.7–98.1)	0.594
p/f ratio 24 h after follow-up; median (IQR)	9	232 (145.3–295.5)	0.767
pO_2_ 3–6 weeks after follow-up; median (IQR) [mmHg]	7	60.4 ((51.4–75.3)	0.128
p/f ratio 3–6 week after follow-up; median (IQR)	7	244.8 (230.5–300)	0.398
pCO_2_ before start; median (IQR) [mmHg]	12	41.8 (39.1–46.7)	
pCO_2_ 2 h after follow-up; median (IQR) [mmHg]	12	42.9 (38.9–49.2)	0.433
pCO_2_ 24 h after follow-up; median (IQR) [mmHg]	9	40.3 (39–48.8)	0.953
pCO_2_ 3–6 weeks after follow-up; median (IQR) [mmHg]	7	40.5 (36.1–47.1)	0.612
Follow-up visit; n (%)	12	7/12 (58.3%)	
lethality during surveillance; n (%)	12	2/12 (16.7%)	

* *p*-value in Wilcoxon signed ranked test for paired samples; alpha = 0.05.

## Data Availability

The datasets used and analyzed in the current study are available from the corresponding author on reasonable request.
